# Enhanced Strength–Ductility Synergy Properties in Selective Laser Melted 316L Stainless Steel by Strengthening Grinding Process

**DOI:** 10.3390/ma15207227

**Published:** 2022-10-17

**Authors:** Jinrui Xiao, Tao Zou, Yiteng Zhang, Zhuan Zhao, Gongbin Tang, Xincheng Xie, Zhongwei Liang, Xiaochu Liu

**Affiliations:** 1 Guangdong Engineering Research Centre for Strengthen Grinding and Micro/Nano High-Performance Machining, Guangzhou University, Guangzhou 510006, China; 2 School of Mechanical and Electrical Engineering, Guangzhou University, Guangzhou 510006, China; 3 School of Physics and Materials Science, Guangzhou University, Guangzhou 510006, China; 4 School of Electromechanical Engineering, Guangdong University of Technology, Guangzhou 510006, China

**Keywords:** grain refinement, microhardness, microstructure, residual stress, strengthening grinding process, strength-ductility synergy

## Abstract

Selective laser melted (SLM) 316L stainless steel (SS) has been widely employed in the fields of designing and manufacturing components with complex shapes and sizes. However, the low yield strength, low ultimate tensile stress, and low hardness of SLM 316L SS components hinder its further application. In this work, the strengthening grinding process (SGP) was used to enhance the mechanical properties of SLM 316L SS. The microhardness, residual stress, microstructure, and tensile properties of all the samples were analyzed. The results demonstrate that the SGP induced higher compressive residual stress and microhardness, as well as higher tensile properties. The maximum hardness and residual stress reached 354.5 HV and −446 MPa, respectively, indicating that the SGP resulted in a plastic deformation layer over 150 μm. The possible mechanisms have been discussed in further detail. Compared to the untreated sample, the SGP sample shows a significant improvement in yield strength (YS), ultimate tensile stress (UTS), and elongation (EL), increasing 30%, 25.5%, and 99.1%, respectively. This work demonstrates that SGP treatment could be an efficient approach to simultaneously improving the strength and ductility of the SLM 316L SS, which makes it more suitable for engineering applications.

## 1. Introduction

316L stainless steel (SS) has been widely used in ocean shipping facilities [1], nuclear reactors [2], biomedicine [3], laser powder bed fusion [4], aerospace equipment [5], and medical devices [6], because of its good formability, excellent corrosion resistance, and non-magnetic properties [7]. The additive manufacturing of 316L SS has attracted great attention and interest owing to its benefits in manufacturing complex geometry, sizes, and shapes [8,9], especially in manufacturing aeronautical and astronautical components, such as attitude control power systems, thrust chambers, and gas generators [10].

Selective laser melting is one of the most popular and promising additive manufacturing techniques and has a low energy cost, low swelling rate, and high precision [11,12]. It molds materials layer-by-layer using a laser beam as well as a moving and cooling system to achieve rapid melting and solidification, which is beneficial for producing parts with high density and fine-grained microstructures [13]. Therefore, SLM is usually used for the design and trial-producing of components with complex shapes and sizes, such as the gas nozzle of an aero-engine, the grille rudder of a rocket, and mold cavities [10,14]. The microstructure and mechanical properties of SLM parts are dependent on parameters of powder size, building direction, composition, laser scanning strategy, among others [15,16,17]. In the additive manufacturing process, SLM 316L SS components face some inevitable challenges, such as high tensile residual stress, low yield strength, low ultimate tensile stress, low hardness, and poor wear resistance, which weaken their mechanical properties and limit their engineering applications [18,19]. Therefore, a suitable post-process must be employed in SLM 316L SS parts to obtain high mechanical properties.

To tackle these issues, many solutions have been proposed, such as shot peening, surface coating, rolling, and water jet peening [20,21,22,23]. Rolling processing offers a high-efficiency solution for generating high-strength layers on the surface of metallic materials that is simple to operate and inexpensive [24]. However, due to the fixed champing mode and severe deformation, it is not suitable for machining components with complex shapes and low strength, as it may lead to serious structural changes [25,26]. The water jet peening process has the advantage of machining complex components with good mechanical properties [27]. However, it requires ultra-high pressure of 40 to 100 MPa, which imposes higher requirements for pressurizing devices. Moreover, it easily leads to surface flaking when it is excessively treated [28]. 

As an alternative, peening techniques can introduce high-level residual compressive stress, microhardness, grain refinement, and strength to the steel surface, and thus are an ideal technique for the post-process of SLM 316L SS [27,29]. Due to its benefits in reliability and environmental adaptability [30], laser shot peening is the most widely used peening method for enhancing the mechanical properties of SLM 316L SS components. Nevertheless, some negative effects, such as oxidization, high-temperature stain, and high cost, are induced during the processing [31,32]. Conventional shot peening (SP) can increase residual compressive stresses, dislocation density, and grain refinement in the subsurface of materials [33]. However, if it is treated improperly, the surface roughness intensifies, which results in the peeling and folding of the treated workpiece [28,34]. Ghosh et al. conducted a surface mechanical attrition treatment (SMAT) for SLM 316L SS at a frequency of 30 kHz with steel balls of 4.75 mm for a duration of 15 min [35]. They obtained excellent strength and ductility, which were introduced by the gradient nanostructure, in SML 316L SS. Similar results were also presented by Portella et al. after SMAT with a 2 mm steel ball for a duration of 15 min at a frequency of 20 kHz [36]. Therefore, SMAT treatment seems to be an efficient technique for improving the strength of SLM 316L SS. However, according to the above studies, the strength–ductility tradeoff obtained with SLM 316L SS remains to be overcome. Additionally, SMAT requires high-frequency vibration for a relatively long time, which results in high energy cost and low treatment efficiency in the selected engineering application. 

The strengthening grinding process (SGP), which employs a mixed abrasive that consists of a strengthening liquid, ceramic balls, and brown corundum powder to impact the surface of the target samples, is an efficient approach for obtaining high mechanical and material properties. The mixed abrasive is driven by the compressed gas or an ultrasonic actuator. Recent evidence suggests that the grain refinement, microhardness, and misorientation density of the treated surfaces can be significantly increased via the impact of the mixed abrasive (diameter less than 1 mm) for a short time, such as 3 min [37,38]. Increasing the grain refinement, microhardness, and misorientation density will also be accompanied by compressive residual stress, which is believed to be beneficial to improving the mechanical properties [39]. Hence, the SGP, which has high treatment efficiency and low energy cost, opens up the possibility of improving the mechanical properties in engineering applications. However, the effect of the SGP on the strength and ductility of SLM 316L SS has not been studied so far.

It is difficult to simulate the entire particle peening process due to the millions of impacts that may be involved and the difficulty of converging them [40]. Especially, the peening of mixed abrasives in the SGP is more complex. Furthermore, many practical boundary conditions of the SGP cannot be fixed accurately during computational simulation. Therefore, this study focuses on the experimental study of SLM 316L SS. The SGP is employed to enhance the mechanical properties of SLM 316L SS. SLM 316L SS is also treated by SP and used for the control sample. Combined with microhardness, residual stress, microstructure, and fracture morphology analysis, the mechanical properties of each group were analyzed, and the mechanism of the strength–ductility synergy enhancement of the SLM 316L SS was revealed.

## 2. Materials and Methods

### 2.1. Materials

The 316L SS samples used in this study were fabricated by SLM. Commercial 316L SS powder with a size range from 15 μm to 53 μm was used for the selective laser melting processing. The chemical composition of the 316L SS powder is illustrated in Table 1.

### 2.2. Additive Manufacturing Procedure

**Figure 1:**
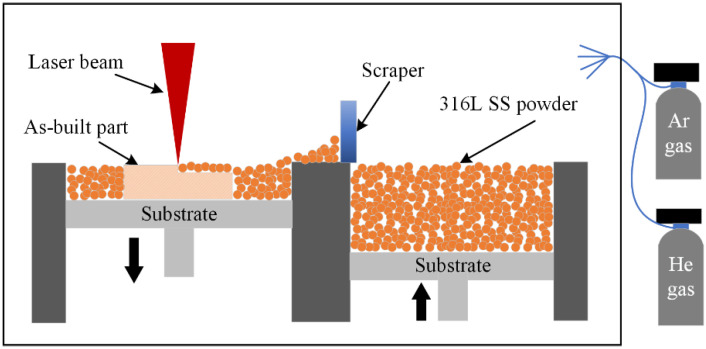
The schematic of SLM processing.

Selective laser melt equipment (UM180, Suzhou Rongzhi 3D Technology Co., Ltd., Suzhou, China) with a spot size of 75 μm was used in this study. In the equipment, a gas comprising Ar and He was produced as the shielding gas, which was purged into the laser-scanned area, as shown in Figure 1. A 316L cube with the dimensions of 50 mm × 50 mm × 50 mm was prepared. The detailed processing parameters of the additive manufacturing process are given in Table 2.

### 2.3. Strengthening Grinding Processing Procedure

**Figure 2:**
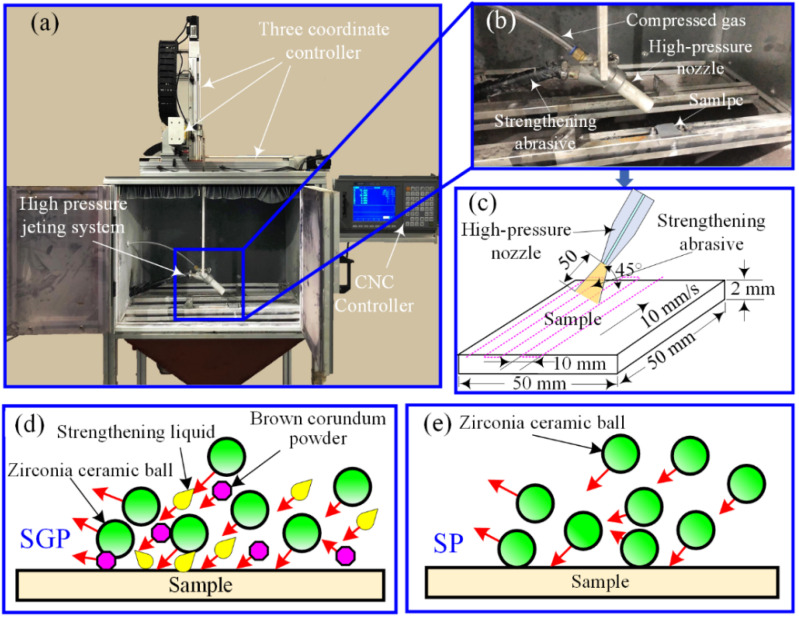
(**a**) Schematic of the strengthening processing equipment, (**b**) high-pressure jetting system, (**c**) processing procedure, (**d**) SP treatment, and (**e**) SGP processing.

**Figure 3:**
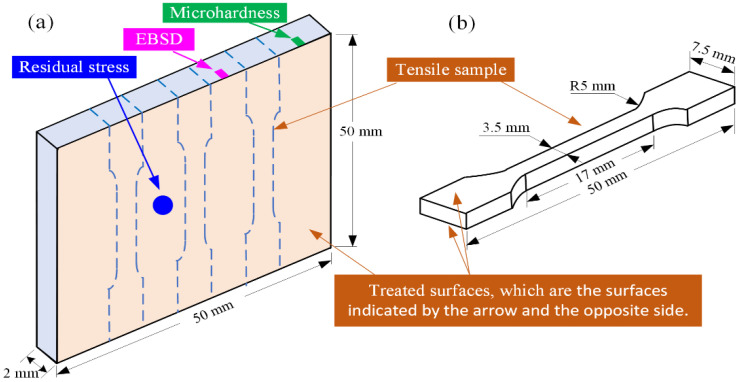
(**a**) Schematic of test process and (**b**) the geometrical parameters of the tensile sample.

Three plates with the dimensions 50 mm × 50 mm × 2 mm were cut off from the processed 316L cube by a wire electric discharge machine (DK7732, Taizhou Wenzhong CNC Equipment Co., Ltd., Taizhou, China). After that, both sides of the three plates were ground by emery paper (grit size ranging from 200 to 1200) resulting in an average surface roughness of approximately 0.5 μm. Prior to further treatment, all the plates were cleaned with alcohol through an ultrasonic bath. Two of them underwent surface strengthening treatment (i.e., SGP or SP), while another did without surface treatment for experimental comparison. Both the SGP and SP treatments were conducted using a homemade strengthening device (see Figure 2a,b). The main working mechanism is that the compressed gas accelerated the strengthening abrasive through a venturi effect, which was then impacted onto the surface of the thin plate through the high-pressure nozzle, as shown in Figure 2c,d. The strengthening abrasive was composed of zirconia ceramic balls, brown corundum powder, and strengthening liquid [34,36], while in the SP treatment, only zirconia ceramic balls were used. The working parameters of the SP and SGP treatment in this work are shown in Table 3. Both the top and the bottom surfaces of the prepared samples were treated by SP or SGP, respectively (See Figure 3). The sample without surface treatment was named the untreated sample, while the other two samples subjected to the SGP and SP processes were named sample SGP and sample SP, respectively.

### 2.4. Residual Stress and Microhardness Observation 

The residual stresses were measured using an EDGE X-ray stress tester (G.N.R. S.r.l. Inc., Milan, Italy), the accuracy of which was ±6.9 MPa. The scan angle ranged from –40° to 40°, with a step of 0.20°. The operating voltage and current were 30.7 kV and 0.083 mA, respectively. Measurements were carried out using a collimator with a diameter of 2 mm and a chromium (Cr) tube with a diffraction plane of <211>. Electrolytic polishing equipment (Struers LectrolPol-5, Struers Inc., Copenhagen, Denmark) was utilized to peel off the surface of the prepared samples layer by layer. The average thickness of each peeled layer is 20 µm, with a total thickness of 200 µm peeled off. The residual stresses were measured three times after each delamination, and the mean value was calculated. A vickers hardness tester (HV-1000, Shanghai optical instrument factory, Shanghai, China), which had an error of less than 5%, was used to test the microhardness from the treated surface to a depth of 140 µm, with a step of 20 µm. The loading force was set to 0.5 N and held for 10 s. Five points were chosen at each depth for measurement, and the average value for the final microhardness was calculated.

### 2.5. Characterization of Microstructure and Mechanical Properties

For the convenience of measurement, three blocks with geometrical dimensions of 5 mm × 5 mm × 2 mm were taken from the prepared samples by electrical discharge wire cutting, followed by mechanical vibration and ultrasonic cleaning. The kernel average misorientation distributions and microstructure were measured by electron back-scattered diffraction (EBSD; Oxford Symmetry Nordly max3, Oxford Instruments, Abingdon, UK). The detected area was 700 μm × 500 μm with a depth of 5 μm below the treated surface (see Figure 2a). The scan step was fixed at 0.8 µm. To investigate the initial microstructure of the additively manufactured 316L SS, a smaller detected area of 420 µm × 290 µm at the sub-topmost level was selected. 

**Figure 4:**
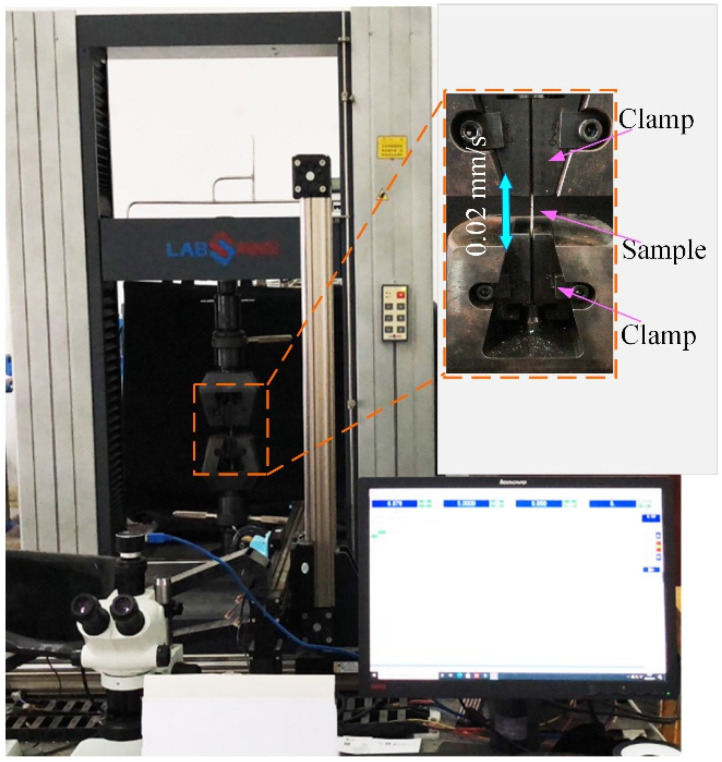
The tensile equipment and test parameters.

Three tensile samples were cut off from each processed thin plate, and their geometry is shown in Figure 2b. An electronic universal testing machine (TSE105D, Wance Technologies Ltd., Shenzhen, China) was used to measure the mechanical properties of the samples, as shown in Figure 4. The accuracies of the tensile speed control, tension detection, and displacement monitoring were ±0.002 mm/s, ±5 N, and ±0.001 mm, respectively. The tensile tests were carried out at room temperature, with a strain rate of 0.02 mm/s, and the average values of YS, UTS, and EL were calculated. 

## 3. Results and Discussion

### 3.1. Mechanical Properties

**Figure 5:**
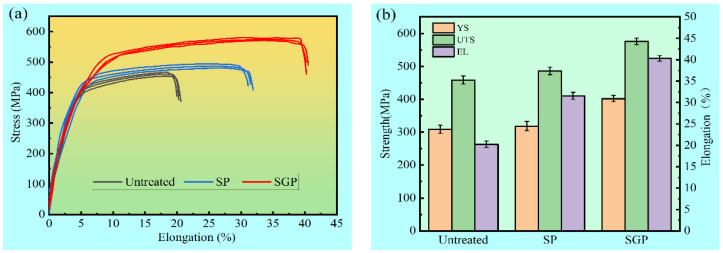
The tensile test results of SGP, SP, and untreated samples: (**a**) the stress–strain curves and (**b**) comparison of YS, UTS, and EL of the samples.

The stress–strain curves of the 316L SS samples fabricated by the different treatments are depicted in Figure 5a, and the values of YS, UTS, and EL are shown in Figure 5b. It is clear that the untreated sample shows the worst tensile properties. Compared to the untreated sample, the YL, UTS, and EL of the SP sample increased by 3.1%, 6%, and 56%, respectively. Interestingly, it can be observed from Figure 3b that the YS (401.7 MPa), UTS (575.6 MPa), and EL (40.3%) of the SGP sample are significantly higher than those of the SP and untreated samples. The YS, UTS, and ductility of the SGP sample are improved by 30%, 25.5%, and 99.1%, respectively, exhibiting the best tensile properties.

### 3.2. Hardness and Residual Stress Evolution of Samples

**Figure 6:**
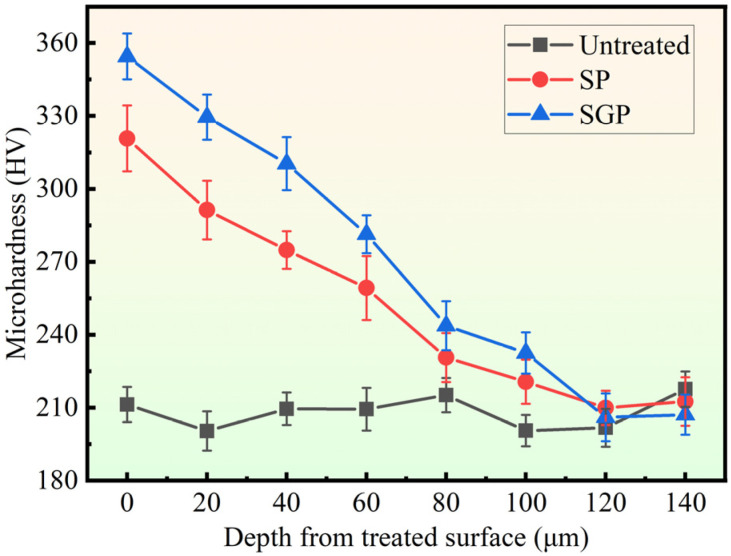
The microhardness distribution in the depth direction of the untreated, SP, and SGP samples.

The average microhardness evolution from the treated surface to a depth of 140 μm was reported in Figure 6. The microhardness of all three samples decreases with the increase in depth. For the SGP sample, the highest microhardness at almost every depth is observed compared to the untreated and SP samples. Furthermore, a significant increase in microhardness is found in the SP and SGP samples compared to the untreated samples. The average surface microhardness of the SGP sample is 354.5 HV, which is 143.1 HV and 33.7 HV higher than that of the untreated and SP samples, respectively. In addition, the mixed strengthening abrasives, comprising zirconia ceramic balls, brown corundum powder, and strengthening liquid, used in the SGP treatment were more beneficial for improving the impact kinetic energy and contest stress than those zirconia ceramic balls that were used in the SP treatment [41]. Therefore, a higher microhardness is obtained in the SGP sample for the reason that the SGP treatment exhibits a stronger work hardening capacity than that of the SP treatment.

**Figure 7:**
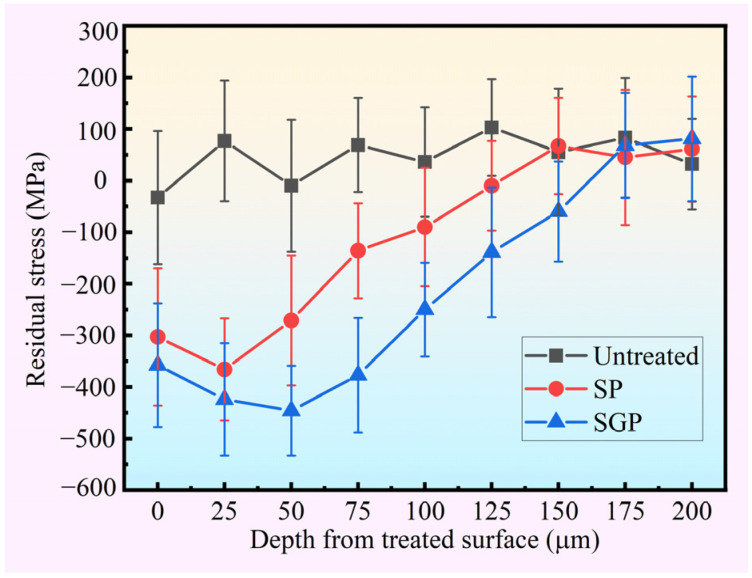
The residual stress distribution in the depth direction of the untreated, SP and SGP samples.

The sample average residual stress as a function of the distance between the surface and the substrate is reported in Figure 7. The residual stress of the untreated sample remains at a stable value of approximately 50 MPa, while a significant difference appears in the SP and SGP samples. They all show compressive residual stress when the depth from the treated surface is less than 150 μm. However, in general, a similar changing tendency is observed: the compressive residual stress value of the SP and SGP samples initially increases slightly, then approximately linearly declines and transforms to a stable tensile stress of 0 to 100 MPa. A maximum average compressive residual stress value of −446 MPa is observed in the SGP sample, which represents an increase of 21.9% and 12.5 times compared to the SP and untreated sample, respectively. Additionally, the compressive residual stress layer of the SGP sample is 25 μm thicker than that of the SP sample. In the SGP treatment, the contact transforms from ball–plate to make the following types of compound contact: abrasive–plate, ball–abrasive–plate, and ball–plate, compared to the SP treatment. Therefore, the contact stress will be increased in the SGP treatment for the purpose of reducing the contact area. The degree of plastic deformation will then be increased compared to that of the SP treatment [42]. Finally, the compressive residual stress is increased following the degree of plastic deformation. Hence, the compressive residual stress value and the layer thickness of the SGP sample are higher than those of the SP sample. 

### 3.3. Reasons for Improvement of Strength and Ductility Synergy Properties of SGP Samples

**Figure 8:**
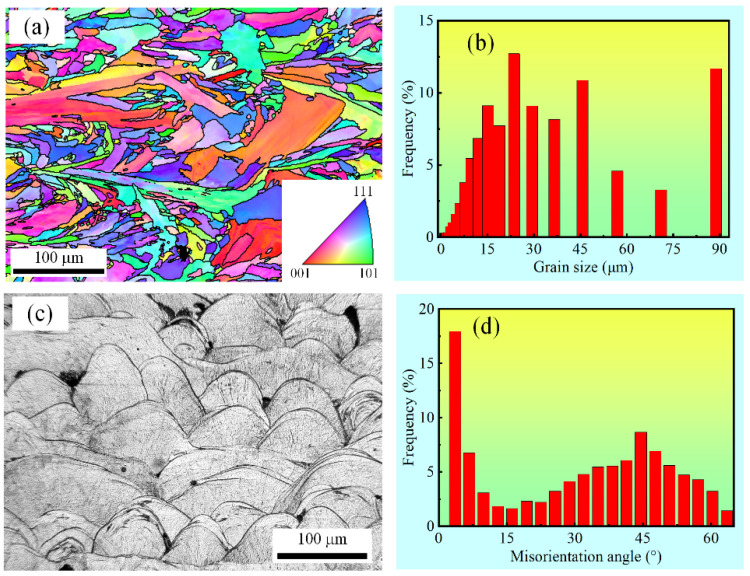
The microstructure, grain size, and misorientation angle distribution of the untreated sample: (**a**) the IPF map, (**b**) grain size analysis, (**c**) the optical micrograph, and (**d**) misorientation angle statistics.

The results of the EBSD and optical micrograph of the untreated sample are presented in Figure 8. An elongated grain morphology is found in the untreated samples due to epitaxial growth. Moreover, a large number of irregular fine grains are found between the coarse long grains. According to Figure 8a, the grains mainly grow along the directions of <001> and <111>. The misorientation angle is mainly concentrated between 30° and 60°, as shown in Figure 8d. A maximum grain size of 90 μm is observed while the others mainly fall in the range from 15 μm to 45 μm (See Figure 8b). The grains of the 316L SS will grow epitaxially in the crystalline orientation along the maximum temperature gradient direction and form an elongated morphology during the SLM process [43]. Semi-elliptical melt pools, which are induced by the layer-by-layer building strategy during the SLM process, are observed (See Figure 8c). To avoid the appearance of holes, the depth of the melt pools was set to be deeper than that of the layer thickness. As a result, the newly formed melt pools cover parts of the previous layer, and the semi-elliptical melt pools form along the laser scan direction. 

**Figure 9:**
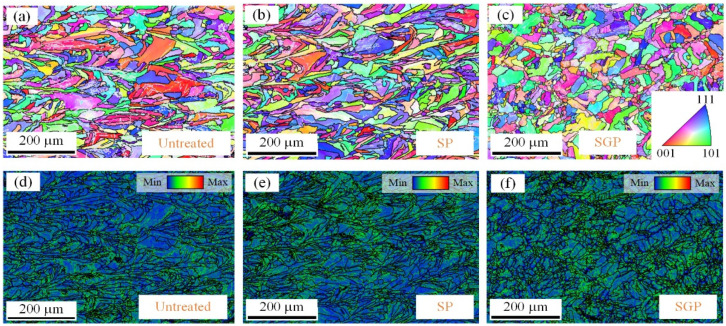
EBSD analyses of the IPF maps and KAM maps of the samples’ cross-sections: (**a**–**c**) crystal orientations of the untreated, SP, and SGP samples. (**d**–**f**) Kernel average misorientation distributions of the untreated, SP, and SGP samples.

Compared to the untreated and SP samples, more grains grew along the <101> direction in the SGP sample. This result also indicates that the grain refinement and lattice distortion introduced by the mixed strengthening abrasive impact are more severe than those induced by the impact of the zirconia ceramic balls alone. Therefore, a more severe deformation and harder surface layer can be obtained in the SGP sample, which is consistent with the previous results in Figure 6. Figure 9d–f reveals the kernel average misorientation of each sample. Obviously, more misorientations are observed in the SP and SGP samples. At the same time, a small amount of misorientation is found in the untreated sample, which was probably introduced by the SLM cooling process. Notably, a more uniform and denser kernel average misorientation network forms in the SGP sample, which is the primary reason for observing a higher compressive residual stress in the SGP sample.

During the SP and SGP treatments, the strength and ductility of the samples are simultaneously improved because of improvements in the dislocation density improvement and back-stress hardening mechanisms introduced by plastic and energy absorption [44]. For the untreated sample, the adhesion ability of the microstructure is poor because of the half-arc stack, which affects the ductility. The mixed strengthening abrasives have higher impact kinetic energy and lower elastic energy than those of the zirconia ceramic balls. As a result, the microhardness of the SGP sample is higher than that of the SP sample. According to the previous studies, the hardness of the metal materials is proportional to their strength [45,46,47]. Hence, the YS and UTS of the SGP sample are higher than those of the SP sample. Unlike the SP treatment, the form of contact in the impact zone changes from single ball–plate contact to mixed ball–plate and microparticle–plate contact during the SGP treatment. Then, the contact stress distribution in the impacted area of the SGP treatment is more uniform. Therefore, a more uniform dislocation network in the plastic deformation zone forms, which in turn makes it more beneficial for improving the ductility of the SGP sample.

**Figure 10:**
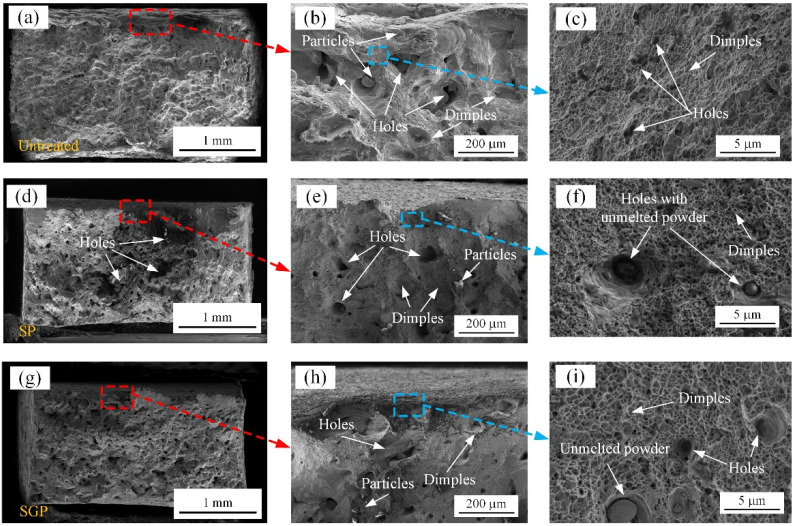
Fracture morphologies of the samples that undergo different processes, (**a**,**d**,**g**), which are the fracture microstructures of the untreated, SP, and SGP samples, respectively. (**b**,**c**) are the magnified views of the untreated sample; (**e**,**f**) are the magnified views of the SP sample; and (**h**,**i**) are the magnified views of the SGP sample.

Figure 10 reports the fracture morphologies of the untreated, SP, and SGP samples. As shown in Figure 10a,d,g, the fracture area of the untreated sample is larger than that of the surface-treated samples. The smallest fracture area is found in the SGP sample, indicating that the largest plastic strain occurred during the tensile process. Meanwhile, there are many dimples, particles, and holes distributed at the edge of the fracture surfaces of all the samples (see Figure 10b,e,h), indicating that ductile fracture occurred. Microcavities, which can be interpreted as the large deformation that occurred during the tensile process, gradually formed in the samples. Over time, these microcavities will rapidly coalesce and tear, finally becoming dimples, holes, and particles. Notably, some large holes were observed in the untreated and SP samples, which were the most vulnerable to crack initiation and brittle fracture [48]. Additionally, some unmelted powders were also observed in these samples (See Figure 10c,f,i), which were introduced by the SLM process. It can be observed that deeper and larger dimples with a more uniform size were formed at the edge of the SGP sample compared to the untreated and SP samples. This result further explains why the SGP sample possesses better strength–ductility synergy properties. In other words, the strengthened layer induced by SPG treatment was proven to be more beneficial to improve the tensile properties of the samples.

### 3.4. Comparison Analysis of Strength and Ductility Properties

**Figure 11:**
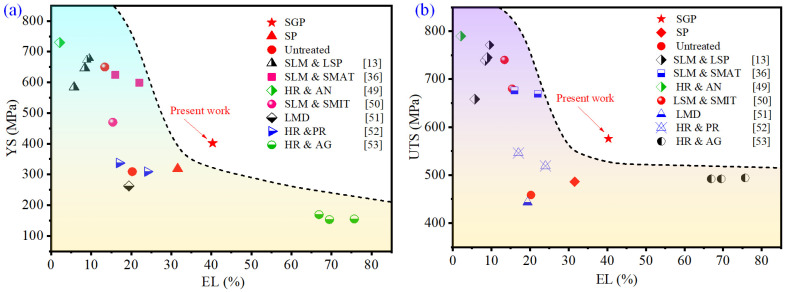
The comparison of the results from the current study and 316L steel modified by different strengthening methods [13,36,49,50,51,52,53]. (**a**) the comparison of YS; (**b**) the comparison of UTS.

Comparisons of the tensile properties in this work with previous research are shown in Figure 11 and Table 4. It is clear to see that SGP induces a better strength-ductility synergy property. Compared to the sample that was processed by SLM and laser shot peening [13], SLM and SMAT [36], hot rolling and annealing [49], and SLM and surface mechanical impact treatment (SMIT) [50], the SGP sample shows outstanding ductility, with an EL value of 40.3%. When compared to the samples subjected to laser metal deposition [51], hot rolling and pressing [52], and SP as well as to those that were untreated, the sample treated by the SGP treatment exhibited higher strength and ductility. Additionally, the SGP sample exhibits advantages in YS and UTS compared to the samples fabricated by hot rolling and aging [53]. The main reason can be concluded as the refinement of grains and the dislocation network induced by SGP treatment resulting in high compressive residual stress, microhardness, strength, and ductility, thus showing good tensile properties. It is worth pointing out that a synergistic enhancement in strength and ductility was found in the SGP samples, while previous studies mentioned above did not. Moreover, the diameter of the mixed abrasive used for SGP treatment was less than 1.5, and the processing time was fixed as 1 min, much larger and longer than in the above previous studies. Hence, this investigation offers a promising solution to overcome the longstanding tradeoff of strength–ductility for engineering applications. However, there are some limitations that deserve study in the future, such as structural changes occurring at a greater depth than the changes in microhardness or residual stresses, which may also affect the tensile properties. Additionally, further study of the evolutionary trends of the cracks in different samples during the tensile test is also of interest.

## 4. Conclusions

In this study, the effects of the SP and SGP treatments on the mechanical properties of SLM 316L SS were investigated. Another sample without treatment was employed for experimental comparison. The results demonstrated that the SGP sample shows the highest microhardness and compressive residual stress, exhibiting values of 354.5 HV and −446 MPa, respectively. Minimum and irregular grains concentrated at 10–35 μm were also obtained in the SGP sample. Additionally, excellent strength-ductility synergy performance was obtained in the SGP sample. Compared to the untreated sample, the YS, UTS, and EL of the SGP sample were remarkably improved by 30%, 25.5%, and 99.1%, respectively. These results could be attributed to the high energetic impact of the mixed abrasives, which consisted of zirconia ceramic balls, strengthening liquid, and brown corundum powder, on the machined surface. Additionally, the grain refinement and misorientation network were formed. Finally, high microhardness and compressive residual stress were formed in the surface layer while simultaneously improving strength and ductility. This study demonstrated that the SGP treatment is a candidate to overcome the longstanding strength–ductility tradeoff preventing the engineering applications of SLM 316L SS. However, there are also several interesting avenues for future research, such as whether the structural changes, which occur at a greater depth than the changes in microhardness or residual stresses, affect the tensile properties and whether differences in the cracks of untreated, SP, and SGP samples occur and evolve during tensile testing.

## Figures and Tables

**Table 1:** Chemical composition of 316L SS powder (in mass fraction, wt%).

Cr	Ni	Mo	Mn	Si	C	P	S	O	Fe
16.84	10.51	2.84	1.03	0.55	0.016	0.013	0.07	0.048	Bal.

**Table 2:** The parameters of the 316L SS additive manufacturing process.

Parameters	Value
Laser power (W)	500
Printing speed (mm/s)	14.3
Preheating temperature (°C)	45
Interlayer temperature (°C)	≤100
Dry extension (mm)	12–16
Inter-channel offset (mm)	3.5

**Table 3:** The technological parameters for strengthening grinding processing.

Parameters	Value
Diameter of zirconia ceramic ball (mm)	1.5
Grain size of brown corundum (μm)	15
Jet pressure (MPa)	0.6
Processing time (s)	60
Jet angle (°)	45
Jet distance (mm)	50
Abrasive flow rate (Kg/min)	10

**Table 4:** Processing and tensile properties of 316 L stainless steel obtained from previous works and present work.

Processing ^a^	YS (MPa)	UTS (MPa)	EL (%)	Refs.
SLM + SGP 1 min	401.7	575.6	40.3	Present work
SLM + SP 1 min	318.5	486.2	31.6	Present work
SLM	309	458.5	20.3	Present work
SLM (BD 0)	582	632	7.7	[13]
SLM (BD 0) & LSP	584	658	5.7	[13]
SLM (BD 45) & LSP	646	739	8.4	[13]
SLM (BD 90) & LSP	679	771	9.5	[13]
SLM & SMAT 10 min	599	669	22	[36]
SLM & SMAT 30 min	624	676	16	[36]
HR + 850 °C/1 h/AN	730	790	2.1	[49]
SLM & SMIT 60 min	470	680	15	[50]
SLM & SMIT 120 min	650	740	16	[50]
LMD	262.3	443.4	19.4	[51]
HR & PR-0.20C	337	546	17	[52]
HR & PR-0.06C	309	519	24	[52]
HR & 1000 °C/AN & 700 °C/1 h/AG	153	492	69.5	[53]
HR & 1000 °C/AN-2Cu	155	494	75.7	[53]
HR & 1000 °C/AN-4Cu	169	492	66.9	[53]

^a^ AN (annealed), AG (aging), BD (build directions), HR (hot rolling), LSP (laser shot peening), LMD (laser metal deposition), PR (pressing), SLM (selective laser melting), SMAT (surface mechanical attrition treatment), SMIT (surface mechanical impact treatment).

## Data Availability

The data presented in this study are available in the article.
